# X-Rays and Hepatic Abscesses

**Published:** 1908-07-04

**Authors:** 


### X-RAYS AND HEPATIC ABSCESSES.

Hepatic abscesses may be divided into two main classes—those which are small and multiple, and those that are larger and few, or even single. It is of the latter that we would speak here—notably of the so-called tropical or dysenteric abscess of the liver.

The treatment for the condition is clearly to evacuate the pus, either by incision and drainage, or by the insertion of a large trocar carrying with it a drainage tube, without incision, according to the Manson method. In either case the first thing required is that the abscess should not merely be diagnosed, but also accurately located beforehand. Hitherto localisation has chiefly been by needling the liver in various directions, the direction of the needling being guided partly by the symptoms and partly by the physical signs. This has often necessitated the insertion of a needle into a good many different places before pus is found; and sometimes there has been failure to hit off the pus with the needle at all, although it has been present all the time.

It is of great importance, therefore, to know that the *x*-rays may afford very valuable assistance in demonstrating the position of such an abscess of the liver, and in indicating the precise direction in which the needle should be passed in order to find the pus at once. Major C. B. Lawson, of the Royal Army Medical Corps, records the following consecutive cases in which the method was used with great success, the needle, guided by the *x*-rays, striking the pus in each case at the first puncture:—

Case I.—A private who had been invalided from Pekin for debility. He gave a history of dysentery, pyrexia, and sweating. His blood showed a leucocytosis of 20,000 per cub. mm., and his liver was greatly enlarged. Screening under the *x*-rays showed a shadow about the size of the fœtal head in the centre of the region of the right lobe of the liver, the lower part of the shadow appearing on a level with the eighth rib in the right mammary line. The patient was examined only in the recumbent position because he was too ill to sit up. His diaphragm was motionless on the right side. An aspirator needle was inserted in the seventh right intercostal space in the anterior axillary line. Pus was found at once. Two inches of the seventh right rib were resected, and a pint and a half of characteristic tropical liver abscess pus were evacuated.

Case II.—A sapper, in whom the diagnosis was hepatitis, or abscess. Screening showed a small shadow near the upper limit of the hepatic dulness on the right side. The aspirator needle was inserted in the mid-axillary line in the ninth right intercostal space, and directed towards the position of the shadow. Four ounces of reddish-brown pus were evacuated.

Case III.—A staff-sergeant, who was admitted to hospital with the diagnosis: Hepatitis, or right-sided pleurisy, or hepatic abscess. The first screening showed fluid in the pleural sac. This fluid became absorbed in due course, and on a second screening a dome-shaped shadow was visible, encroaching on the right chest. The diaphragm on this side had an excursion of about half an inch. The dome-shaped swelling could not be made out by the ordinary methods of clinical examination. The patient had a tender spot one and a half inches below and external to the right nipple. The aspirator needle was inserted here, and directed towards the dome-shaped shadow, pus being struck at a depth of three inches. A portion of the eighth rib between the nipple and the anterior axillary lines was removed, and a large drainage tube inserted. Five ounces of thick curdy pus were drawn off.

The *x*-rays would only be used, naturally, after all the more usual clinical methods had been employed first. It is clear, however, that there are many cases of hepatic abscess in which the *x*-rays discover more than other methods can without them; not only do they assist in diagnosing and locating the abscess, but they help in deciding whether or not there are more abscesses than the one in the same liver. All the above were single abscesses, and all made good recoveries.

